# Associations Between Micronutrient Status, Hormones, and Immune Status During Pregnancy and Child Growth in Rural Bangladesh: A Prospective Cohort Study

**DOI:** 10.1016/j.cdnut.2025.107596

**Published:** 2025-11-08

**Authors:** Belinda Chen, Andrew N Mertens, Chih-Hsien Lin, Sophia T Tan, Farheen Jamshed, Diego Figueroa, Caitlin Hemlock, Zachary Butzin-Dozier, Lia CH Fernald, Christine P Stewart, Alan E Hubbard, Md Ziaur Rahman, Shahjahan Ali, Benjamin F Arnold, Firdaus S Dhabhar, Douglas A Granger, Mahbubur Rahman, Stephen P Luby, John M Colford, Audrie Lin

**Affiliations:** 1 School of Public Health, University of California Berkeley, Berkeley, CA, United States; 2 Division of Infectious Diseases and Geographic Medicine, Stanford University, Stanford, CA, United States; 3 Department of Environmental and Occupational Health Sciences, University of Washington, Seattle, WA, United States; 4 Institute for Global Nutrition, University of California Davis, Davis, CA, United States; 5 Department of Microbiology and Environmental Toxicology, University of California, Santa Cruz, Santa Cruz, CA, United States; 6 Department of Epidemiology, Colorado School of Public Health, University of Colorado, Denver, CO, United States; 7 Francis I. Proctor Foundation, University of California San Francisco, San Francisco, CA, United States; 8 Department of Psychiatry & Behavioral Sciences, Department of Microbiology and Immunology, Sylvester Comprehensive Cancer Center, Miller School of Medicine, University of Miami, Miami, FL, United States; 9 Institute for Interdisciplinary Salivary Bioscience Research, University of California, Irvine, Irvine, CA, United States; 10 Department of Pediatrics, Johns Hopkins University School of Medicine, Baltimore, MD, United States; 11 Environmental Health and WASH, Health System and Population Studies Division, International Centre for Diarrhoeal Disease Research, Bangladesh, Dhaka, Bangladesh; 12 Global Health and Migration Unit, Department of Women’s and Children’s Health, Uppsala University, Uppsala, Sweden

**Keywords:** child growth outcomes, maternal micronutrients, maternal hormones, maternal inflammation, pregnancy

## Abstract

**Background:**

Poor growth in early childhood is associated with increased mortality, impaired cognitive development, and reduced adult economic productivity, which may result in higher risks of social immobility and intergenerational poverty.

**Objective:**

We aimed to evaluate whether maternal hormones, immune status, and micronutrient status during all trimesters of pregnancy were associated with child growth outcomes in the first two years after birth.

**Methods:**

This observational study used data collected from the WASH Benefits trial in rural Bangladesh to examine associations between maternal hormones [plasma cortisol, estriol], immune status [C-reactive protein, α-1-acid glycoprotein (AGP), cytokine sum score], and micronutrient status [vitamin D (25-hydroxy-D [25(OH)D]), ferritin, soluble transferrin receptor, retinol binding protein (RBP)] during pregnancy and subsequent measures of child growth. Length-for-age z-score (LAZ), weight-for-length z-score (WLZ), and insulin-like growth factor 1 (IGF-1) at 3, 14, and 28 mo were measured as the primary outcomes. All outcomes were adjusted for confounding variables, and the *P* values were adjusted using the Benjamini–Hochberg procedure. We used generalized additive models, adjusted for covariates, and reported the mean difference in outcomes between the 25th and 75th percentiles of the exposure distribution.

**Results:**

In the adjusted models of this study (*n* = 636), at 3 mo of age, maternal AGP and RBP were positively associated with infant WLZ. By 14 mo, higher maternal estriol was linked with higher LAZ, and RBP remained positively associated with WLZ. At 28 mo, maternal estriol showed a negative association with IGF-1, and a higher cytokine sum score was negatively associated with WLZ.

**Conclusions:**

These findings suggest the possible pathways through which maternal biomarkers influence early childhood growth, highlighting the intrauterine environment’s critical role in shaping developmental outcomes.

The parent trial was registered at clinicaltrials.gov (NCT01590095).

## Introduction

Poor growth in early childhood remains a persistent challenge in low- and middle-income countries (LMICs), contributing to increased mortality [1,2], increased risk of infectious disease outcomes [3,4], and reduced adult productivity [5,6]. Thus, under Sustainable Development Goal 2, which aims to end hunger and improve nutrition, linear growth faltering within the first 2 y of life is recognized as a critical factor impacting early childhood development [7,8]. Linear growth faltering remains prevalent in LMICs despite decades of investment in child nutrition interventions, suggesting that factors beyond postnatal nutrition contribute to poor growth outcomes [7]. Although the mechanisms underlying poor child growth are not yet fully understood, emerging evidence suggests that the in-utero environment, including maternal stress, undernutrition, dysregulated hormones, and systemic inflammation, may play a central role in child growth outcomes.

Maternal inflammation during pregnancy may contribute to child stunting through disruption of the growth hormone insulin-like growth factor 1 (IGF-1) axis [9]. Previous studies demonstrate that proinflammatory cytokines, including IL-1β, IL-6, and TNF-α, directly suppress the hepatic IGF-1 gene expression and increase production of IGF-1 binding proteins, reducing IGF-1 bioavailability and limiting skeletal growth [[9], [10], [11]]. These findings are supported by observational studies that confirmed inverse associations between maternal inflammatory markers and IGF-1 concentrations in the mother and child [9,12,13]. C-reactive protein (CRP), synthesized in response to acute inflammation, and α-1-acid glycoprotein (AGP), which indicates chronic systemic inflammation, serve as established biomarkers of maternal inflammatory status during pregnancy [[14], [15], [16], [17], [18]]. Maternal proinflammatory cytokines cross the placenta and elevate fetal cytokine concentrations, potentially leading to persistent growth dysfunction [19].

Maternal stress during pregnancy can elevate fetal exposure to cortisol, which can impair growth by disrupting IGF-1 signaling, limiting placental nutrient transfer, and inhibiting immune development, all of which lead to intrauterine growth restriction [20]. Cortisol-mediated pathways operate through the hypothalamic-pituitary-adrenal axis, causing proinflammatory cytokine release that crosses the placenta and creates a persistent inflammatory in-utero environment [20,21]. Chronic cortisol levels in pregnant females create compounding adverse conditions that may contribute to long-term growth deficits that extend through infancy and childhood.

Beyond regulating fetal cortisol exposure, the placenta serves as an important endocrine organ producing hormones essential for fetal growth. Estriol (E3), a key estrogen involved in pregnancy and fetal growth, is synthesized through a pathway that requires both fetal and placental input. Because E3 is derived from fetal sources, its levels serve as a sensitive marker of overall intrauterine function. Adequate E3 promotes uteroplacental blood flow, supports skeletal development, and helps regulate gestational length [22]. In contrast, low maternal E3 concentrations have been linked to pregnancy loss and adverse growth outcomes [22]. Together, the stress and hormone pathways demonstrate how low E3 levels combined with elevated cortisol can interfere with healthy fetal development.

Beyond hormonal regulation, maternal micronutrient status is a critical determinant of fetal development and long-term health. Vitamin D, a fat-soluble vitamin transferred across the placenta in the form of 25-hydroxyviatmin D [25(OH)D], is essential for modulating placental function, calcium homeostasis, and bone mineralization [23]. Iron is equally important in development, as it supports hemoglobin synthesis and oxygen delivery in fetuses to prevent premature delivery [24,25]. Vitamin A is also critical for proper fetal development by regulating cell differentiation through its active metabolite, retinoic acid, which is essential for skeletal development [26]. Collectively, deficiencies in these critical micronutrients compromise placental transfer of essential nutrients and disrupt growth processes [[27], [28]]. These findings further highlight the intricate interplay between maternal health, placental function, and early childhood growth, reinforcing the need for comprehensive prenatal nutrition to support child growth outcomes.

Despite growing evidence linking prenatal conditions to child growth outcomes, comprehensive analyses examining multiple biomarkers simultaneously are limited. Previous studies have typically studied biomarkers in isolation, limiting the understanding of how various maternal and prenatal factors interact to individually shape the in-utero environment. Additionally, most research has examined outcomes only at birth or in early infancy, providing incomplete insights into how prenatal exposures affect growth across the first 2 y of life when growth velocity is highest.

This study aimed to examine the individual associations between a diverse panel of maternal biomarkers including hormones, inflammatory markers, and micronutrient status during pregnancy and subsequent child growth measures. We hypothesized that elevated maternal cortisol and inflammation would lead to poor child growth outcomes, whereas adequate micronutrient status and higher gestational E3 levels would lead to enhanced growth.

## Methods

### Study site and design

This observational analysis used data collected from the WASH Benefits Bangladesh trial, which was conducted from May 2012 to March 2016 in rural villages located in the Gazipur, Kishoreganj, Mymensingh, and Tangail districts of Bangladesh [29,30]. The WASH Benefits trial was a large, cluster-randomized controlled trial designed to test whether household-level water quality, sanitation, handwashing, and nutrition interventions either alone or in combination, could improve child growth and reduce enteric disease. Eligible pregnant females were geographically grouped in clusters of 8 households and block randomized into one of 7 arms: chlorinated drinking water (water); upgraded sanitation (sanitation); promotion of handwashing with soap (handwashing); combined water, sanitation, and handwashing (WSH); counseling on appropriate child nutrition and lipid-based nutrient supplements (nutrition); combined nutrition, water, sanitation, and handwashing (N+WSH), and control (data collection only). For these analyses, mother–child dyads were assessed from the environmental enteric dysfunction substudy of the WASH Benefits Bangladesh study, which included the control, WSH, nutrition, and N+WSH arms [31,32]. This dataset has been used to investigate a range of maternal and child health outcomes; this study focuses on growth-related measures, including length-for-age Z-score (LAZ), weight-for-length Z-score (WLZ), and IGF-1 concentrations.

### Participants

**FIGURE 1:**
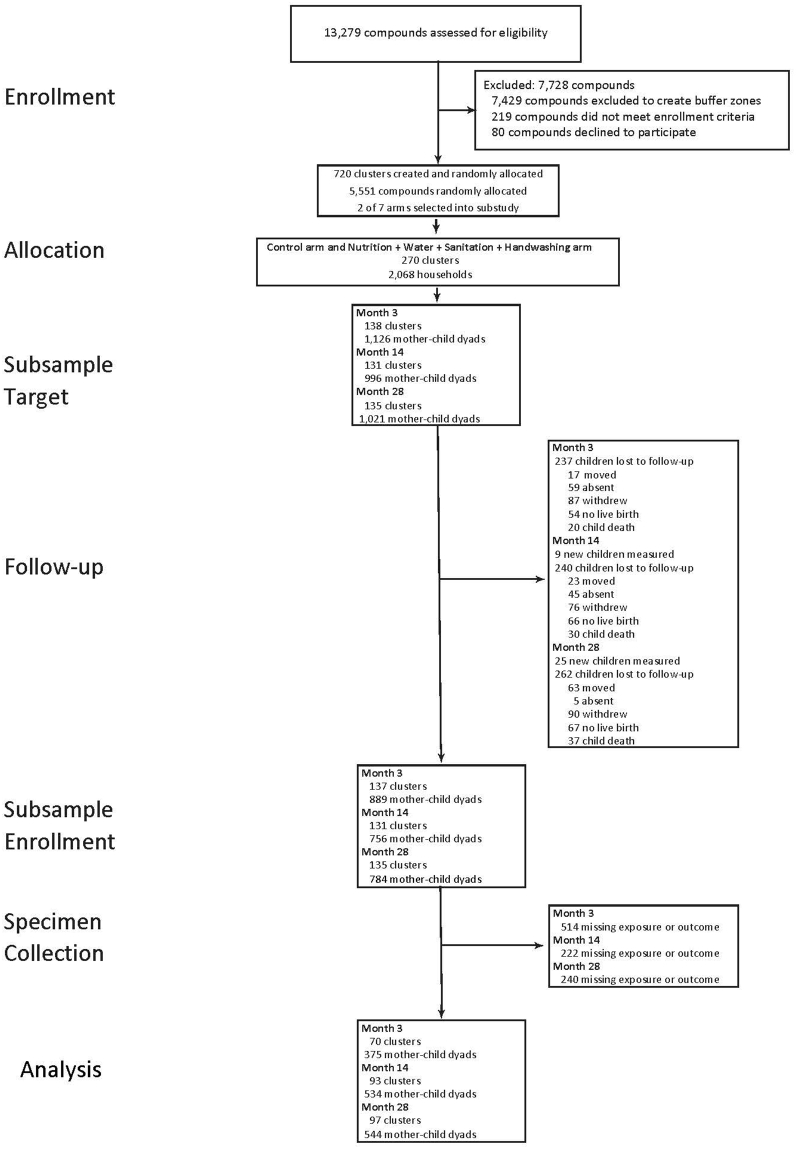
Enrollment characteristics flow chart of participant status throughout enrollment, data collection, and statistical analysis.

In the parent WASH Benefits Bangladesh trial, 5551 pregnant females and their in-utero children in 720 clusters across Gazipur, Kishoreganj, Mymensingh, and Tangail were enrolled in their first or second trimester between May 2012 and July 2013 [29,30]. Inclusion criteria included intent to reside within the study village for the next 2 y, whereas exclusions included anticipated relocation within 1 y, lack of home ownership, or high iron concentrations in household water. Within this trial, the environmental enteric dysfunction (EED) substudy recruited a subset of mother–child dyads for additional biological sample collection at 3, 14, and 28 mo [31]. The present study focuses on the 636 dyads within the EED substudy that had both maternal biomarker measurements during any trimester of pregnancy and subsequent child growth outcomes available (Figure 1). If a mother gave birth to twins, both children were enrolled in the study.

**TABLE 1:** Enrollment characteristics.

		*n* (%) or median (P25, P75)^1^
Child	Female	311 (51%)
Anthropometry (3 mo)	Length-for-age *Z*-score	−1.28 (−2.01, −0.54)
	Weight-for-age *Z*-score	−1.22 (−1.9, −0.51)
	Weight-for-length *Z*-score	−0.26 (−1.17, 0.47)
	Head circumference-for-age *Z*-score	−1.94 (−2.5, −1.14)
Anthropometry (14 mo)	Length-for-age *Z*-score	−1.46 (−2.23, −0.82)
	Weight-for-age *Z*-score	−1.41 (−2.08, −0.8)
	Weight-for-length *Z*-score	−1 (−1.67, −0.32)
	Head circumference-for-age *Z-*score	−1.81 (−2.42, −1.22)
Anthropometry (28 mo)	Length-for-age *Z*-score	−1.62 (−2.36, −1.01)
	Weight-for-age *Z*-score	−1.58 (−2.15, −0.97)
	Weight-for-length *Z*-score	−0.99 (−1.6, −0.36)
	Head circumference-for-age *Z*-score	−1.78 (−2.37, −1.24)
Diarrhea (14 mo)	Caregiver-reported 7-d recall	84 (16%)
Diarrhea (28 mo)	Caregiver-reported 7-d recall	43 (8%)
Acute respiratory illness (14 mo)	Caregiver-reported 7-d recall	162 (31%)
Acute respiratory illness (28 mo)	Caregiver-reported 7-d recall	132 (24%)
Mother	Age (y)	24 (20, 27)
	Gestational age (wk)	21.86 (17.29, 25.86)
Anthropometry at enrollment	Height (cm)	149.93 (146.7, 153.74)
Education	Schooling completed (y)	6 (4, 9)
Depression (14 mo)	CESD-20 score	10 (6, 16)
Depression (28 mo)	CESD-20 score	9 (5, 17)
Perceived stress (28 mo)	Perceived stress scale score	14 (11, 18)
Intimate partner violence	Any lifetime exposure	1 (0, 1)
Household		
Household food insecurity	Food-insecure households	161 (28%)

Abbreviation: CESD-20, Center for Epidemiological Studies Depression Scale Revised.

1 P25, P75, 25th percentile, 75th percentile.

### Data collection and procedures

Maternal and household characteristics, such as education level, household food insecurity, and distance to primary drinking water source, were assessed during the first or second trimester of pregnancy. In a follow-up visit across all trimesters, trained phlebotomists collected 10 mL of venous blood from each pregnant female using trace metal-free certified needles and tubes (Sarstedt). Samples were placed immediately on ice, transported the same day to the field laboratory, processed, and stored at −80°C until analysis.

For child assessments in the EED substudy, child anthropometry measurements and venipunctures were collected at 3, 14, and 28 mo. At each of these visits, 5 mL of venous blood was collected from infants and children by trained technicians. Child samples were processed and stored using the same protocols as the maternal samples.

### Laboratory methods

The following exposure measurements were assessed: maternal serum E3 and cortisol, maternal immune status {maternal serum CRP, serum AGP, 13 plasma cytokines [IL-1β, IL-6, TNF-α, IL-2, IL-12p70, interferon-γ (IFN-γ), IL-4, IL-5, IL-13, IL-17A, IL-21, IL-10, granulocyte-macrophage colony-stimulating factor (GM-CSF)]}, and maternal nutritional status [maternal serum vitamin D (25-hydroxy-D [25(OH)D]), ferritin, soluble transferrin receptor (sTfR), retinol binding protein (RBP), vitamin A deficiency, vitamin D deficiency, and iron deficiency].

Maternal hormones were measured at the International Centre for Diarrhoeal Disease Research, Bangladesh (icddr,b) laboratory in Mymensingh, Bangladesh. Following kit protocols, the IBL-America Free Estriol ELISA was used to measure maternal serum E3. The DetectX Cortisol Immunoassay kit was used to measure maternal serum cortisol. The coefficient of variation for the cortisol and E3 assays was <10%. Because biomarker distributions were skewed, E3 and cortisol were log-transformed for analysis.

Maternal serum AGP and CRP were measured at the VitMin Lab following previously published procedures [32].

The panel of 13 plasma cytokines (IL-1β, IL-6, TNF-α, IL-2, IL-12p70, IFN-γ, IL-4, IL-5, IL-13, IL-17A, IL-21, IL-10, GM-CSF) was measured using multiplex Luminex technology (Millipore kit HSTCMAG-28SK) at the University of Maryland. Detailed procedures have been previously published [32]. Cytokine calculations were performed with Bio-Rad Bio-Plex Software. The coefficient of variation for the multiplex Luminex assay was <20%. Because all the distributions of immune markers were skewed, cytokines, CRP, and AGP values were log-transformed.

The icddr,b Nutritional Biochemistry Lab in Dhaka, Bangladesh, measured maternal serum vitamin D (25-hydroxy-D [25(OH)D]) using the Roche Kit. Vitamin D was measured by electrochemiluminescence binding assay using the Roche automated immunoanalyzer (cobas e601). Maternal serum ferritin, sTfR, and RBP were measured using the sandwich ELISA assay and were log-transformed for analysis (VitMin Lab). Maternal serum vitamin D was analyzed on the original scale, whereas ferritin, sTfR, and RBP were log-transformed due to skewed distributions. These nutritional biomarkers were assessed as continuous and binary exposures defined as follows: vitamin D deficiency (25-OH-VitD < 30 nmol/L), iron deficiency (ferritin < 12 μg/L or sTfR > 8.3 mg/L), and vitamin A deficiency (RBP < 0.70 μmol/L). The coefficient of variation for these assays were <10%. Because ferritin, sTfR, and RBP are affected by the acute-phase response, we corrected these values for CRP and AGP using the Biomarkers Reflecting Inflammation and Nutritional Determinants of Anemia (BRINDA) method [33]. Although more recent BRINDA guidance suggests that adjusting RBP for inflammation may not be necessary when estimating vitamin A deficiency in adult females, the correction was still applied in this study to minimize potential bias from acute-phase responses when assessing associations with child growth outcomes.

Child plasma IGF-1 concentrations were measured via the Quantikine ELISA kit (R&D Systems, Inc.) at the icddr,b laboratory in Mymensingh, Bangladesh. Out-of-range samples were analyzed again at higher or lower dilutions. The coefficient of variation for the IGF-1 assay was <10%.

### Construction of cytokine sum score

Each of the 13 log-transformed cytokine values was scaled to generate *Z*-scores. Because all the cytokine *Z*-scores were observed to be proinflammatory and of similar magnitude through calculations of the Pearson coefficient, the Z-scores were summed to create a composite score representing overall systemic inflammation [34]. To minimize bias from missing data, missing immune markers were imputed using the k-nearest neighbors algorithm.

### Anthropometry measurements

Child anthropometry outcomes were measured at ages 3, 14, and 28 mo. Following standard protocols for anthropometric outcome measurements, pairs of trained anthropometrics measured recumbent length (accurate to 0.1 cm), weight without clothing, and head circumference in triplicate. Following standardized protocols [[35], [36],^37^], pairs of trained staff members recorded anthropometry measurements. The median of the 3 measurements was used to calculate LAZ and WLZ standardized to the WHO 2006 child growth standards (https://www.who.int/tools/child-growth-standards/software) [37]. Child age was calculated based on birthdates, which were confirmed when feasible using vaccination cards. We excluded children from *Z-*score calculations if their growth measurements fell outside biologically reasonable ranges as per WHO guidelines [37]. The median of the 3 measurements was used to calculate LAZ and WLZ.

### Statistical approach

The analysis plan, including hypotheses, rationale, and statistical methods, was registered on Open Science Framework (https://osf.io/djbsh/) prior to accessing the data. All analyses were conducted in R (version 4.4.1). Data and scripts are publicly available on Open Science Framework and GitHub.

To account for potential nonlinearity in the relationship between maternal exposures and child growth outcomes, we utilized natural smoothing splines to summarize the mean LAZ, WLZ, and IGF-1 of children across the distributions of maternal cortisol, E3, immune status, and nutrition status. Generalized additive models were unadjusted and adjusted for confounders such as child age, sex, and prescreened covariates that were found to be significantly related to the outcome in bivariate analyses (*P* < 0.2) [28]. The prescreening process used the likelihood ratio test, and only covariates meeting the *P* < 0.2 threshold were included in the adjusted models. Covariates with low variability in the study population (prevalence <5%) were excluded. Unadjusted results can be found in Supplementary Materials (Supplementary Tables 1–4). Cortisol results were adjusted using the time the sample was placed on the cold chain as a proxy for time of day, thus controlling for the cortisol awakening response. A detailed list of prescreened covariates can be found in the analysis plan on Open Science Framework (https://osf.io/djbsh/).

Due to the exploratory nature of these observational analyses, the focus was on the overall strength and consistency of the associations rather than on isolated *P* values. Specifically, we examined whether multiple biomarkers within the same biological pathway (e.g., inflammation, micronutrients) were associated with child growth outcomes consistently in the same direction. When such consistency was observed, this pattern was interpreted as suggestive of an underlying biological relationship. In contrast, when the associations lacked consistency across related biomarkers, the individual statistically significant findings were interpreted with caution, noting the possibility of spurious results due to multiple testing. We also applied the Benjamini–Hochberg procedure to every comparison in the analysis to correct for multiple testing by controlling the false discovery rate (FDR), using a threshold of <0.1 for significance.

### Ethics

The parent trial was registered at clinicaltrials.gov (NCT01590095) and overseen by a data safety monitoring committee convened by icddr,b. The study protocols were approved by human subjects committees at icddr,b (PR-11063 and PR-14108), the University of California, Berkeley (2011-09-3652 and 2014-07-6561), and Stanford University (25863 and 35583). Participants and primary caregivers of all children provided written informed consent.

## Results

### Enrollment characteristics

The parent WASH Benefits trial enrolled 5551 pregnant females and their children. This substudy enrolled 375 children at age 3 mo, 534 children at age 14 mo, and 544 children at age 28 mo (Figure 1). A total of 636 mother–child dyads had pregnancy and child biomarker measurements at 3 mo, 14 mo, and 28 mo.

At sample collection, the median age of the females was 24 y (IQR: 20–27; Table 1), and the median estimated gestational age of the children was 22.7 wk (IQR: 16.1–27.9). The females completed a median of 6 y of education (IQR: 4–9). 57% of enrolled females (288) reported having exposure to intimate partner violence in their lifetime. 162 households (28%) were also reported to be food-insecure on the Household Food Insecurity Access Scale [38].

For the children enrolled in the substudy, at 3 mo the median LAZ was −1.29, weight-for-age Z-score (WAZ) was −1.21, WLZ was −0.26, and the head circumference-for-age *Z*-score was −1.83 (Table 1). At 14 and 28 mo, LAZ and WAZ decreased (Table 1).

### Maternal hormones and child growth

**FIGURE 2:**
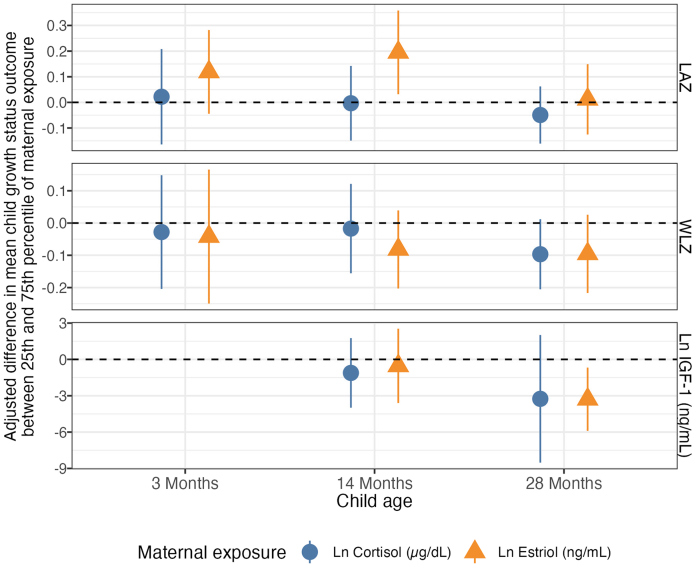
Adjusted associations between maternal hormones during pregnancy and child growth at 3, 14, and 28 mo. Adjusted differences in mean child LAZ score, WHZ score, and IGF-1 between 25th and 75th percentiles of maternal estriol and cortisol measurements. Adjusted for the prespecified and prescreened covariates: child sex, child birth order, child gestational age, mother’s age, mother’s height, mother’s education, household food security, number of children aged <18 y in the household, number of people living in the compound, distance (in minutes) to the primary water source, household materials (wall, floor, roof), asset-based household wealth (electricity, wardrobe, table, chair or bench, khat, chouki, working radio, working black/white or color television, refrigerator, bicycle, motorcycle, sewing machine, mobile phone, land phone, number of cows, number of goats, number of chickens), and maternal exposure to intimate partner violence (IPV) during pregnancy and lifetime. IGF-1, insulin-like growth factor 1; LAZ, length-for-age *Z*-score; WLZ, weight-for-length *Z*-score.

After adjusting for prespecified covariates and controlling for FDR, a positive association was observed between maternal E3 levels and child LAZ at 14 mo {+0.2 SD adjusted difference between the 25th and 75th percentile [95% confidence interval (CI): 0.03, 0.36; FDR-corrected *P* value = 0.07; Figure 2, Table 2, Supplementary Table 1]}. Higher E3 levels in the mother were also associated with reductions in IGF-1 at 28 mo [−3.29 log μg/L adjusted difference (95% CI: −5.9, −0.68), FDR-corrected *P* value = 0.03]. There were no observed associations between maternal E3 and child WLZ (Table 2, Supplementary Table 1) or between maternal cortisol and child growth outcomes (Table 3, Supplementary Table 2).

**TABLE 2:** Association between maternal estriol during pregnancy and child growth outcomes at 3, 14, and 28 mo.

Maternal estriol and child growth status^2^	Outcome	*N*	25th percentile	75th percentile	Outcome, 75th percentile vs. 25th percentile				FDR-corrected *P* value
					Predicted outcome at 25th percentile	Predicted outcome at 75th percentile	Coefficient (95% CI)	*P* value	
Ln estriol (ng/mL)	Length-for-age *Z*-score 3 mo	303	1.04	1.81	−1.41	−1.29	0.12 (−0.04, 0.28)	0.15	0.23
	Weight-for-length *Z*-score 3 mo	301	1.04	1.78	−0.19	−0.23	−0.04 (−0.25, 0.17)	0.71	0.71
	Length-for-age *Z*-score 14 mo	508	0.76	1.71	–0.65	−0.45	0.2 (0.03, 0.36)	0.02^1^	0.07^1^
	Weight-for-length *Z*-score 14 mo	504	0.74	1.71	−0.83	−0.91	−0.08 (−0.2, 0.04)	0.19	0.28
	Ln IGF-1 14 mo (μg/L)	417	0.73	1.71	38.79	38.25	−0.53 (−3.6, 2.53)	0.75	0.75
	Length-for-age *Z*-score 28 mo	533	0.78	1.73	−1.45	−1.44	0.01 (−0.13, 0.15)	0.87	0.87
	Weight-for-length *Z*-score 28 mo	536	0.78	1.73	−0.91	−1.00	−0.1 (−0.22, 0.03)	0.12	0.28
	Ln IGF-1 28 mo (μg/L)	499	0.78	1.74	53.57	50.28	−3.29 (−5.9, −0.68)	0.01	0.03^1^

*N*, 25th percentile, and 75th percentile are from the adjusted analyses.

Abbreviations: CI, confidence interval; FDR, false discovery rate; IGF-1, insulin-like growth factor 1.

1 *P* < 0.1 after adjusting for multiple comparisons using the Benjamini–Hochberg procedure.

2 Adjusted for the prespecified and prescreened covariates: child sex, child birth order, child gestational age, mother’s age, mother’s height, mother’s education, household food security, number of children aged <18 y in the household, number of people living in the compound, distance (in minutes) to the primary water source, household materials (wall, floor, roof), asset-based household wealth (electricity, wardrobe, table, chair or bench, khat, chouki, working radio, working black/white or color television, refrigerator, bicycle, motorcycle, sewing machine, mobile phone, land phone, number of cows, number of goats, number of chickens), and maternal exposure to intimate partner violence during pregnancy and lifetime.

**TABLE 3:** Associations between maternal plasma cortisol during pregnancy and child growth outcomes at 3, 14, and 28 mo.

Maternal plasma cortisol and child growth status^1^	Outcome	*N*	25th percentile	75th percentile	Outcome, 75th percentile vs. 25th percentile				FDR-corrected *P* value
					Adjusted				
					Predicted outcome at 25th percentile	Predicted outcome at 75th percentile	Coefficient (95% CI)	*P* value	
Ln Cortisol (μg/dL)	Length-for-age *Z*-score 3 mo	303	2.66	3.29	−1.33	−1.30	0.02 (−0.16, 0.21)	0.83	0.99
	Weight-for-length *Z*-score 3 mo	336	2.66	3.29	−0.27	−0.30	−0.03 (−0.2, 0.15)	0.77	0.82
	Length-for-age *Z*-score 14 mo	508	2.51	3.26	−1.13	−1.13	0 (−0.15, 0.14)	0.97	0.99
	Weight-for-length *Z*-score 14 mo	504	2.52	3.27	−0.86	−0.88	−0.02 (−0.16, 0.12)	0.82	0.82
	Ln IGF-1 14 mo (μg/L)	424	2.46	3.26	39.16	38.05	−1.12 (−3.99, 1.76)	0.45	0.45
	Length-for-age *Z*-score 28 mo	533	2.53	3.27	−1.41	−1.46	−0.05 (−0.16, 0.06)	0.39	0.99
	Weight-for-length *Z*-score 28 mo	535	2.53	3.27	−0.94	−1.04	−0.1 (−0.21, 0.01)	0.08	0.24
	Ln IGF-1 28 mo (μg/L)	498	2.53	3.26	51.84	48.59	−3.25 (−8.52, 2.01)	0.23	0.45

*N*, 25th percentile, and 75th percentile are from the adjusted analyses.

Abbreviations: CI, confidence interval; FDR, false discovery rate; IGF-1, insulin-like growth factor 1.^a^

1 Adjusted for prespecified and prescreeened covariates: the covariates mentioned in Table 2.

### Maternal inflammation and child growth

After adjustment for covariates, the association between maternal AGP and child WLZ at 3 mo was positive [+0.38 SD adjusted difference between the 25th and 75th percentiles (95% CI: 0.07, 0.69), FDR-corrected *P* value = 0.07]. The maternal sum score of 13 cytokines was negatively associated with child WLZ at 28 mo [−0.1 SD adjusted difference (95% CI: −0.14, −0.07), FDR-corrected *P* value < 0.01].

**FIGURE 3:**
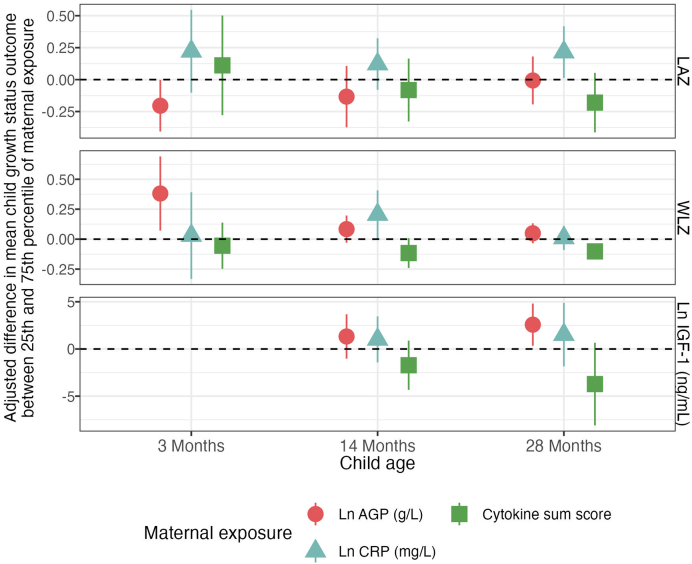
Adjusted associations between maternal immune status during pregnancy and child growth at 3, 14, and 28 mo. Adjusted differences in mean child LAZ score between 25th and 75th percentiles of maternal CRP, AGP, and 13-cytokine sum score measurements. Adjusted for the prespecified and prescreened covariates: child sex, child birth order, child gestational age, mother’s age, mother’s height, mother’s education, household food security, number of children aged <18 y in the household, number of people living in the compound, distance (in minutes) to the primary water source, household materials (wall, floor, roof), asset-based household wealth (electricity, wardrobe, table, chair or bench, khat, chouki, working radio, working black/white or color television, refrigerator, bicycle, motorcycle, sewing machine, mobile phone, land phone, number of cows, number of goats, number of chickens), and maternal exposure to intimate partner violence (IPV) during pregnancy and lifetime. AGP, α-1-acid glycoprotein; CRP, C-reactive protein; IGF-1, insulin-like growth factor 1; LAZ, length-for-age *Z*-score; WLZ, weight-for-length *Z*-score.

Elevated log CRP was associated with higher values across all child growth outcomes but was not significant after FDR correction (Figure 3, Table 4, Supplementary Table 3). The positive associations between log CRP and growth outcomes were consistent with positive associations found between AGP and WLZ.

**TABLE 4:** Associations between maternal inflammation during pregnancy and child growth outcomes at 3, 14, and 28 mo.

Maternal inflammation and child growth status^2^	Outcome	*N*	25th percentile	75th percentile	Outcome, 75th percentile vs. 25th percentile				FDR-corrected *P* value
					Adjusted				
					Predicted outcome at 25th percentile	Predicted outcome at 75th percentile	Coefficient (95% CI)	*P* value	
Ln AGP (g/L)	Length-for-age *Z*-score 3 mo	307	−1.11	−0.6	−1.2	−1.4	−0.2 (−0.41, 0)	0.05	0.31
	Weight-for-length *Z*-score 3 mo	340	−1.11	−0.59	−0.45	−0.07	0.38 (0.07, 0.69)	0.02	0.07^1^
	Length-for-age *Z*-score 14 mo	513	−1.11	−0.54	−0.85	−0.98	−0.13 (−0.37, 0.11)	0.28	0.64
	Weight-for-length *Z*-score 14 mo	509	−1.11	−0.54	−0.91	−0.82	0.08 (−0.03, 0.2)	0.15	0.27
	Ln IGF-1 14 mo (μg/L)	428	−1.14	−0.54	38.74	40.07	1.32 (−1.03, 3.67)	0.27	0.41
	Length-for-age *Z*-score 28 mo	538	−1.11	−0.58	−1.4	−1.41	−0.01 (−0.19, 0.18)	0.95	0.82
	Weight-for-length *Z*-score 28 mo	540	−1.12	−0.58	−0.99	−0.94	0.05 (−0.03, 0.13)	0.25	0.38
	Ln IGF-1 28 mo (μg/L)	503	−1.14	−0.58	50.95	53.52	2.58 (0.34, 4.81)	0.02	0.14
Ln CRP (mg/L)	Length-for-age *Z*-score 3 mo	307	0.12	1.51	−1.39	−1.17	0.22 (−0.1, 0.55)	0.18	0.34
	Weight-for-length *Z*-score 3 mo	340	0.1	1.51	−0.36	−0.33	0.03 (−0.33, 0.39)	0.88	0.88
	Length-for-age *Z*-score 14 mo	513	0	1.42	−1.14	−1.02	0.12 (−0.08, 0.32)	0.24	0.34
	Weight-for-length *Z*-score 14 mo	509	0	1.43	−1.01	−0.81	0.21 (0, 0.41)	0.05	0.14
	Ln IGF-1 14 mo (μg/L)	428	−0.12	1.42	38.54	39.55	1.01 (−1.42, 3.45)	0.42	0.42
	Length-for-age *Z*-score 28 mo	538	−0.08	1.42	−1.5	−1.29	0.22 (0.01, 0.42)	0.04	0.31
	Weight-for-length *Z*-score 28 mo	540	−0.08	1.42	−0.96	−0.95	0.01 (−0.09, 0.11)	0.85	0.88
	Ln IGF-1 28 mo (ug/L)	503	−0.11	1.41	49.86	51.38	1.52 (−1.84, 4.88)	0.38	0.42
Sum score of 13 cytokines	Length-for-age *Z*-score 3 mo	284	−0.7	0.65	−1.34	−1.23	0.11 (−0.28, 0.5)	0.59	0.64
	Weight-for-length *Z*-score 3 mo	281	−0.7	0.65	−0.28	−0.34	−0.05 (−0.25, 0.14)	0.59	0.76
	Length-for-age *Z*-score 14 mo	421	−0.64	0.63	−1.22	−1.3	−0.08 (−0.33, 0.16)	0.53	0.64
	Weight-for-length *Z*-score 14 mo	421	−0.64	0.63	−0.83	−0.94	−0.12 (−0.24, 0.01)	0.07	0.15
	Ln IGF-1 14 mo (μg/L)	365	−0.63	0.64	38.84	37.11	−1.72 (−4.33, 0.89)	0.2	0.4
	Length-for-age *Z*-score 28 mo	509	−0.64	0.65	−1.27	−1.45	−0.18 (−0.41, 0.05)	0.13	0.34
	Weight-for-length *Z*-score 28 mo	510	−0.64	0.65	−0.86	−0.96	−0.1 (−0.14, −0.07)	<0.01	<0.01^1^
	Ln IGF-1 28 mo (μg/L)	479	−0.64	0.65	50.99	47.27	−3.72 (−8.1, 0.66)	0.1	0.29

*N*, 25th percentile, and 75th percentile are from the adjusted analyses.

Abbreviations: AGP, α-1-acid glycoprotein; CI, confidence interval; CRP, C-reactive protein; FDR, false discovery rate; IGF-1, insulin-like growth factor 1.

1 *P* < 0.1 after adjusting for multiple comparisons using the Benjamini–Hochberg procedure.

2 Adjusted for prespecified and prescreened covariates: the covariates mentioned in Table 2.

### Maternal micronutrients and child growth

**FIGURE 4:**
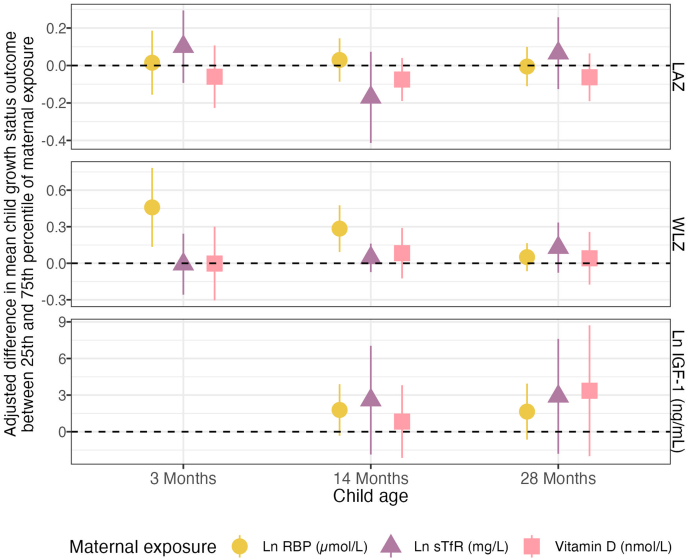
Adjusted associations between maternal micronutrients during pregnancy and child growth at 3, 14, and 28 mo. Adjusted differences in mean child LAZ score between 25th and 75th percentiles of maternal RBP, sTfR, and vitamin D measurements. Adjusted for the prespecified and prescreened covariates: child sex, child birth order, child gestational age, mother’s age, mother’s height, mother’s education, household food security, number of children aged <18 y in the household, number of people living in the compound, distance (in minutes) to the primary water source, household materials (wall, floor, roof), asset-based household wealth (electricity, wardrobe, table, chair or bench, khat, chouki, working radio, working black/white or color television, refrigerator, bicycle, motorcycle, sewing machine, mobile phone, land phone, number of cows, number of goats, number of chickens), and maternal exposure to intimate partner violence (IPV) during pregnancy and lifetime. IGF-1, insulin-like growth factor 1; LAZ, length-for-age Z-score; RBP, retinol binding protein; sTfR, soluble transferrin receptor; WLZ, weight-for-length Z-score.

Elevated maternal log RBP was associated with higher child WLZ at 3 mo [+0.46 SD adjusted difference (95% CI: 0.13, 0.78), FDR-corrected *P* value = 0.05] and 14 mo [+0.28 SD adjusted difference (95% CI: 0.09, 0.48), FDR-corrected *P* value = 0.05]. There were no associations observed between RBP and LAZ or IGF-1 at any of the time points. Furthermore, maternal vitamin A deficiency, vitamin D {25-hydroxy-D [25(OH)D]} deficiency, and sTfR deficiency yielded no significant associations for any of the 3 outcomes at any time point (Figure 4, Table 5, Supplementary Table 4).

**TABLE 5:** Associations between maternal micronutrients during pregnancy and child growth outcomes at 3, 14, and 28 mo.

Maternal micronutrients and child growth status	Outcome	*N*	25th percentile	75th percentile	Outcome, 75th percentile vs. 25th percentile				
					Adjusted^1^				
					Predicted outcome at 25th percentile	Predicted outcome at 75th percentile	Coefficient (95% CI)	*P* value	FDR-corrected *P* value
Vitamin D (nmol/L)	Length-for-age Z-score 3 mo	346	32.62	53.37	−1.25	−1.31	−0.06 (−0.23, 0.11)	0.49	0.67
	Weight-for-length *Z*-score 3 mo	344	32.62	53.47	−0.2	−0.2	0 (−0.31, 0.3)	0.99	0.99
	Length-for-age *Z*-score 14 mo	526	32.59	54.73	−0.89	−0.96	−0.07 (−0.18, 0.04)	0.21	0.48
	Weight-for-length *Z*-score 14 mo	526	32.59	54.73	−0.85	−0.77	0.08 (−0.12, 0.29)	0.44	0.76
	Ln IGF-1 14 mo (μg/L)	431	32.48	54.53	38.98	39.81	0.83 (−2.15, 3.81)	0.6	0.77
	Length-for-age *Z*-score 28 mo	544	32.51	55.34	−1.41	−1.48	−0.06 (−0.19, 0.06)	0.34	0.61
	Weight-for-length *Z*-score 28 mo	544	32.51	55.34	−0.85	−0.81	0.04 (−0.18, 0.26)	0.72	0.95
	Ln IGF-1 28 mo (μg/L)	507	32.59	55.53	51.41	54.77	3.36 (−2, 8.71)	0.22	0.44
									
Vitamin D deficiency^2^	Length-for-age *Z*-score 3 mo	346	0	0	−1.35	−1	0.35 (0, 0.7)	0.05	0.18
	Weight-for-length *Z*-score 3 mo	344	0	1	−0.16	−0.37	−0.21 (−0.57, 0.15)	0.26	0.68
	Length-for-age *Z*-score 14 mo	526	0	0	−0.99	−0.77	0.22 (0.01, 0.43)	0.04	0.17
	Weight-for-length *Z*-score 14 mo	526	0	1	−0.79	−0.83	−0.04 (−0.26, 0.18)	0.74	0.95
	Ln IGF-1 14 mo (μg/L)	431	0	1	38.4	37.34	−1.06 (−5.49, 3.37)	0.65	0.77
	Length-for-age *Z*-score 28 mo	544	0	0	−1.47	−1.26	0.21 (0.03, 0.4)	0.03	0.17
	Weight-for-length *Z*-score 28 mo	544	0	1	−0.92	−0.84	0.08 (−0.11, 0.28)	0.4	0.76
	Ln IGF-1 28 mo (μg/L)	507	0	1	52.53	51.88	−0.65 (−5.67, 4.36)	0.81	0.81
									
Ln RBP (μmol/L)	Length-for-age *Z*-score 3 mo	346	0.14	0.53	−1.3	−1.28	0.01 (−0.16, 0.19)	0.87	0.92
	Weight-for-length *Z*-score 3 mo	344	0.14	0.53	−0.39	0.07	0.46 (0.13, 0.78)	0.01	0.05^3^
	Length-for-age *Z*-score 14 mo	526	0.13	0.52	−0.91	−0.88	0.04 (−0.08, 0.15)	0.56	0.67
	Weight-for-length *Z*-score 14 mo	526	0.13	0.52	−0.92	−0.64	0.28 (0.09, 0.48)	0	0.05^3^
	Ln IGF-1 14 mo (μg/L)	431	0.13	0.52	37.72	39.5	1.78 (−0.33, 3.9)	0.1	0.44
	Length-for-age *Z*-score 28 mo	544	0.12	0.51	−1.41	−1.42	−0.01 (−0.11, 0.1)	0.92	0.92
	Weight-for-length *Z*-score 28 mo	544	0.12	0.51	−0.89	−0.84	0.05 (−0.06, 0.17)	0.4	0.76
	Ln IGF-1 28 mo (μg/L)	507	0.13	0.52	51	52.65	1.65 (−0.65, 3.94)	0.16	0.44
									
Vitamin A deficiency1	Length-for-age *Z*-score 3 mo	346	0	0	−1.28	−1.43	−0.15 (−0.76, 0.47)	0.65	0.73
	Weight-for-length *Z*-score 3 mo	344	0	1	−0.19	−0.19	0 (−0.64, 0.64)	0.99	0.99
	Length-for-age *Z*-score 14 mo	526	0	0	−0.89	−1.06	−0.17 (−0.54, 0.2)	0.38	0.62
	Weight-for-length *Z*-score 14 mo	526	0	1	−0.8	−0.7	0.1 (−0.28, 0.48)	0.62	0.93
	Ln IGF-1 14 mo (μg/L)	431	0	1	39.83	30.84	−8.99 (−17.09, −0.89)	0.03	0.35
	Length-for-age *Z*-score 28 mo	544	0	0	−1.42	−1.68	−0.26 (−0.59, 0.06)	0.11	0.32
	Weight-for-length *Z*-score 28 mo	544	0	1	−0.92	−0.95	−0.03 (−0.36, 0.3)	0.86	0.99
	Ln IGF-1 28 mo (μg/L)	507	0	1	52.23	54.03	1.8 (−7.07, 10.66)	0.7	0.77
Ln sTfR (mg/L)	Length-for-age *Z*-score 3 mo	346	1.3	1.74	−1.34	−1.24	0.1 (−0.09, 0.29)	0.31	0.61
	Weight-for-length *Z*-score 3 mo	344	1.3	1.74	−0.14	−0.15	−0.01 (−0.26, 0.24)	0.96	0.99
	Length-for-age Z-score 14 mo	526	1.29	1.71	−0.73	−0.91	−0.18 (−0.43, 0.06)	0.14	0.36
	Weight-for-length *Z-*score 14 mo	526	1.29	1.71	−0.8	−0.76	0.04 (−0.07, 0.16)	0.46	0.76
	Ln IGF-1 14 mo (μg/L)	431	1.29	1.68	38.12	40.71	2.59 (−1.87, 7.04)	0.26	0.44
	Length-for-age *Z*-score 28 mo	544	1.3	1.71	−1.42	−1.36	0.07 (−0.13, 0.26)	0.51	0.67
	Weight-for-length *Z*-score 28 mo	544	1.3	1.71	−0.87	−0.74	0.13 (−0.08, 0.33)	0.22	0.68
	Ln IGF-1 28 mo (μg/L)	507	1.3	1.7	50.63	53.53	2.9 (−1.81, 7.6)	0.23	0.44
									
Iron deficiency^2^	Length-for-age *Z*-score 3 mo	346	0	0	−1.31	−1.2	0.11 (−0.21, 0.43)	0.52	0.67
	Weight-for-length *Z*-score 3 mo	344	0	1	−0.15	−0.35	−0.2 (−0.54, 0.14)	0.25	0.68
	Length-for-age *Z*-score 14 mo	526	0	0	−0.98	−1.19	−0.21 (−0.4, −0.02)	0.03	0.17
	Weight-for-length *Z*-score 14 mo	526	0	1	−0.79	−1	−0.21 (−0.4, −0.01)	0.04	0.23
	Ln IGF-1 14 mo (μg/L)	431	0	1	39.13	37.25	−1.88 (−5.93, 2.17)	0.37	0.55
	Length-for-age *Z*-score 28 mo	544	0	0	−1.38	−1.61	−0.22 (−0.39, −0.06)	0.01	0.16
	Weight-for-length *Z*-score 28 mo	544	0	1	−0.91	−1.01	−0.1 (−0.27, 0.07)	0.26	0.68
	Ln IGF-1 28 mo (μg/L)	507	0	1	53.21	49.8	−3.41 (−7.93, 1.11)	0.14	0.44

25th percentile, and 75th percentile are from the adjusted analyses.

Abbreviations: CI, confidence interval; FDR, false discovery rate; IGF-1, insulin-like growth factor 1; RBP, retinol binding protein; sTfR, soluble transferrin receptor.

1 Adjusted for prespecified and prescreened covariates: the covariates mentioned in Table 2.

2 For binary exposure variables, 25th and 75th percentiles represent the distribution of the binary exposure (0/1) in the study population, whereas the predicted outcome represents the estimated child growth measurements for the exposure group.

3 *P* < 0.1 after adjusting for multiple comparisons using the Benjamini–Hochberg procedure.

## Discussion

In this substudy of the WASH Benefits Bangladesh randomized controlled trial, we investigated associations between maternal hormones, inflammation markers, and micronutrients to better understand the complex relationships between maternal biomarkers during pregnancy and early child growth outcomes. After adjusting for covariates, we observed several significant relationships. Higher maternal E3 was associated with higher LAZ at 14 mo, but lower IGF-1 levels at 28 mo. Higher maternal AGP levels were associated with higher WLZ at 3 mo, whereas a higher maternal cytokine sum score was associated with lower WLZ at 28 mo. Finally, higher maternal RBP was associated with higher WLZ at 3 mo and 14 mo. These results were not fully consistent across time points or biomarker domains, and given the exploratory nature of this analysis, some of these associations may reflect chance findings despite FDR correction.

Elevated maternal E3 levels were associated with higher LAZ at 14 mo, which is aligned with other studies linking low maternal E3 levels to fetal growth restriction and adverse neonatal outcomes, consistent with E3’s role as a marker of fetoplacental health [21,39]. Higher E3 levels may promote fetal bone growth and placental nutrient transfer by stimulating uteroplacental blood flow, leading to better LAZ outcomes in infants [21]. The inverse association between maternal E3 and child IGF-1 at 28 mo, however, suggests a nuanced relationship between maternal hormones and child growth factors that may reflect the hormonal regulation of the IGF-1 axis. Estrogens suppress the biological activity of growth hormone by reducing Janus kinase 2 (JAK2) phosphorylation, which results in reductions in IGF-1 production [40]. However, this association should be interpreted cautiously, given the inconsistency between growth outcomes and the possibility of a spurious finding.

The finding that maternal AGP and the cytokine sum score also have complex, time-dependent associations with postnatal growth in children is consistent with the emerging understanding of inflammation’s effects on child growth [12]. AGP, an acute-phase protein indicative of systemic inflammation, showed an unexpected positive association with WLZ at 3 mo and IGF-1 at 28 mo, whereas the cytokine sum score was negatively associated with WLZ at 28 mo. One possible explanation for the contradiction is that mild or transient maternal inflammation could enhance placental nutrient transfer, leading to greater fat deposition in early infancy, whereas prolonged inflammation may suppress the IGF-1 axis and impair growth over time [41]. However, given the lack of consistency across the different inflammation outcomes and timepoints, the results may simply demonstrate chance variance rather than true effects.

Maternal RBP was positively associated with infant WLZ, highlighting the known anti-inflammatory properties of vitamin A in supporting child growth [26]. Low concentrations of vitamin A in pregnant females have been linked to poor placental development and impaired immune function, increasing risks of infant morbidity [26]. In this study, however, higher maternal RBP levels may not reflect a direct effect of vitamin A on child growth but instead serve as an indicator of broader maternal nutritional status and health [4]. The association between maternal RBP and infant WLZ was evident when RBP was analyzed as a continuous variable, but not when applying the binary threshold for vitamin A deficiency. This likely reflects the low prevalence of vitamin A deficiency in our sample (7%), which limited statistical power to detect differences. This interpretation suggests that improving maternal micronutrient status overall can have measurable benefits for child growth.

This study had several limitations. The comparison of multiple maternal and child biomarkers over multiple time points increased the possibility of spurious associations due to correlated measurements or biomarker interference. The Benjamini–Hochberg method was utilized to control the false discovery rate, but this method assumes independence or positive dependence between tests. These assumptions may not fully hold in a study of highly interrelated biomarkers and outcomes, and as such, some risk of Type I error remains.

Another key limitation is the timing of the biomarker collection. Maternal biomarkers analyzed in this study were only assessed once during the pregnancy, despite the known fluctuation of immune, hormonal, and micronutrient levels across gestation. This single time point, as well as the infrequent measurement of child growth outcomes, limits the ability to capture temporal patterns or identify critical windows of exposure at higher granularity.

We also examined each biomarker independently rather than using a multivariate approach. Methods such as path analysis may have provided additional insights into interrelated mechanisms, but these were not feasible given the sample size and the limited number of significant associations.

Finally, it is important to recognize that this analysis captures relative variation in growth outcomes within this specific cohort rather than explaining why children in LMICs experience growth faltering. To provide additional context, the median maternal CRP concentration in this cohort was 1.8 mg/L, slightly higher than published medians for healthy pregnant females [0.8 mg/L (95% CI: 0.4, 1.5)], which suggests modestly elevated systemic inflammation in this cohort [42]. Additionally, the WHO defines vitamin D deficiency during pregnancy as serum 25(OH)D < 50 nmol/L, placing this cohort’s median level of 42.1 nmol/L as slightly deficient [43]. In contrast, a Danish pregnancy cohort reported median 25(OH)D levels of 74.7 nmol/L (IQR: 57–92 nmol/L) at 24 wk of gestation, highlighting that vitamin D levels in this cohort are shifted downward compared with high-income settings [44]. Further studies would benefit from comparing outcome distributions directly to external reference populations and adjusting for prior child size between time points.

This study adds to the growing understanding of how early-life exposures shape child health outcomes by examining the roles of hormones, inflammation, and micronutrient status during pregnancy. The findings from this prospective cohort study suggest that these prenatal exposures are associated with growth outcomes in early childhood, potentially operating through time-sensitive biological mechanisms. By exploring multiple pathways in parallel, this study highlights the intrauterine environment as a critical determinant of child development. These results support the hypothesis that optimizing maternal health could be a viable strategy for promoting healthy growth in early childhood. Future research incorporating longitudinal biomarker assessment during pregnancy and analytical approaches will be essential to elucidate the mechanisms underlying healthy growth trajectories.

## Author contributions

The authors’ responsibilities were as follows – BC, ANM, LCHF, CPS, AEH, BFA, FSD, DAG, MR, SPL, JMC, AL: designed research; BC, ANM, C-HL, STT, FJ, DF, CH, ZB-D, LCHF, CPS, AEH, MZR, SA, BFA, FSD, DAG, MR, SPL, JMC, AL: conducted research; BC, ANM: analyzed data or performed statistical analysis, BC, ANM, AL: wrote the article; BC, ANM, AL: had primary responsibility for the final content; and all authors: read and approved the manuscript.

## Funding

This study was supported by the National Institute of Allergy and Infectious Diseases of the National Institutes of Health (grant number K01AI136885 to AL) and Global Development grant OPPGD759 from the Bill & Melinda Gates Foundation to the University of California, Berkeley.

## Conflict of interest

AL and STT received funding for salary through a grant from the National Institute of Allergy and Infectious Diseases of the National Institutes of Health ANM, LCHF, CPS, AEH, MZR, SA, BFA, FSD, MR, SPL, JMC, and AL received funding for either salary or consulting fees through a grant from the Bill & Melinda Gates Foundation for this study. The funder approved the design of the study. However, the funder played no role in data collection, analysis, interpretation, or any decisions related to publication. The corresponding author had full access to all study data and final responsibility around decision-making while submitting for publication. The content is solely the responsibility of the authors and does not necessarily represent the official views of the National Institutes of Health. In the interest of full disclosure, Douglas Granger is the founder and chief scientific and strategy adviser at Salimetrics LLC and SalivaBio LLC, and these relationships are managed by the policies of the committees on conflict of interest at the Johns Hopkins University School of Medicine and the University of California at Irvine.

## References

[1] Black M.M., Walker S.P., Fernald L.C.H., Andersen C.T., DiGirolamo A.M., Lu C. (2017). Advancing early childhood development: from science to scale 1. Lancet.

[2] McDonald C.M., Olofin I., Flaxman S., Fawzi W.W., Spiegelman D., Caulfield L.E. (2013). The effect of multiple anthropometric deficits on child mortality: meta-analysis of individual data in 10 prospective studies from developing countries. Am. J. Clin. Nutr..

[3] Olofin I., McDonald C.M., Ezzati M., Flaxman S., Black R.E., Fawzi W.W. (2013). Associations of suboptimal growth with all-cause and cause-specific mortality in children under five years: a pooled analysis of ten prospective studies. PLOS One.

[4] Black R.E., Victora C.G., Walker S.P., Bhutta Z.A., Christian P., de Onis M. (2013). Maternal and child undernutrition and overweight in low-income and middle-income countries. Lancet.

[5] Adair L.S., Fall C.H., Osmond C., Stein A.D., Martorell R., Ramirez-Zea M. (2013). Associations of linear growth and relative weight gain during early life with adult health and human capital in countries of low and middle income: findings from five birth cohort studies. Lancet.

[6] Fink G., Peet E., Danaei G., Andrews K., McCoy D.C., Sudfeld C.R. (2016). Schooling and wage income losses due to early-childhood growth faltering in developing countries: national, regional, and global estimates. Am. J. Clin. Nutr..

[7] Gil J.D.B., Reidsma P., Giller K., Todman L., Whitmore A., van Ittersum M. (2019). Sustainable development goal 2: improved targets and indicators for agriculture and food security. Ambio.

[8] Martorell R. (2017). Improved nutrition in the first 1000 days and adult human capital and health. Am. J. Hum. Biol..

[9] Prendergast A.J., Rukobo S., Chasekwa B., Mutasa K., Ntozini R., Mbuya M.N. (2014). Stunting is characterized by chronic inflammation in Zimbabwean infants. PLOS ONE.

[10] Witkowska-Sędek E., Pyrżak B. (2021). Chronic inflammation and the growth hormone/insulin-like growth factor-1 axis. Cent. Eur. J. Immunol..

[11] Lazarides C., Epel E.S., Lin J., Blackburn E.H., Voelkle M.C., Buss C. (2019). Maternal pro-inflammatory state during pregnancy and newborn leukocyte telomere length: a prospective investigation. Brain Behav. Immun..

[12] Leviton A., Allred E.N., Fichorova R.N., VanderVeen D.K., O'Shea T.M., Kuban K. (2019). Early postnatal IGF-1 and IGFBP-1 blood levels in extremely preterm infants: relationships with indicators of placental insufficiency and with systemic inflammation. Am. J. Perinatol.

[13] Hellgren G., Löfqvist C., Hansen-Pupp I., Gram M., Smith L.E., Ley D. (2018). Increased postnatal concentrations of pro-inflammatory cytokines are associated with reduced IGF-I levels and retinopathy of prematurity. Growth Horm. IGF Res..

[14] Ballantyne C.M., Nambi V. (2005). Markers of inflammation and their clinical significance. Atheroscler. Suppl..

[15] Vermeire S., Van Assche G., Rutgeerts P. (2005). The role of C-reactive protein as an inflammatory marker in gastrointestinal diseases. Nat. Rev. Gastroenterol. Hepatol..

[16] Ansar W., Ghosh S. (2016). Biology of C-Reactive Protein in Health and Disease.

[17] Mackiewicz A., Mackiewicz K. (1995). Glycoforms of serum α1-acid glycoprotein as markers of inflammation and cancer. Glycoconj. J..

[18] Fournier T., Medjoubi-N N., Porquet D. (2000). Alpha-1-acid glycoprotein. Biochim. Biophys. Acta..

[19] Coussons-Read M.E. (2013). Effects of prenatal stress on pregnancy and human development: mechanisms and pathways, Obstet. Med..

[20] Parker V.J., Douglas A.J. (2010). Stress in early pregnancy: maternal neuro-endocrine-immune responses and effects. J. Reprod. Immunol..

[21] Kaijser M., Granath F., Jacobsen G., Cnattingius S., Ekbom A. (2000). Maternal pregnancy estriol levels in relation to anamnestic and fetal anthropometric data. Epidemiology.

[22] Kowalczyk T.D., Cabaniss M.L., Cusmano L. (1998). Association of low unconjugated estriol in the second trimester and adverse pregnancy outcome. Obstet. Gynecol..

[23] Shin J.S., Choi M.Y., Longtine M.S., Nelson D.M. (2010). Vitamin D effects on pregnancy and the placenta. Placenta.

[24] Scholl T.O., Reilly T. (2000). Anemia, iron and pregnancy outcome. J. Nutr..

[25] Georgieff M.K. (2020). Iron deficiency in pregnancy. Am. J. Obstet. Gynecol..

[26] Bastos Maia S., Rolland Souza A.S., Costa Caminha M.F., Lins da Silva S., Callou Cruz R.S.B.L., Carvalho Dos Santos C. (2019). Vitamin A and pregnancy: a narrative review. Nutrients.

[27] Hovdenak N., Haram K. (2012). Influence of mineral and vitamin supplements on pregnancy outcome. Eur. J. Obstet. Gynecol. Reprod. Biol..

[28] Arnold B.F., Null C., Luby S.P., Unicomb L., Stewart C.P., Dewey K.G. (2013). Cluster-randomised controlled trials of individual and combined water, sanitation, hygiene and nutritional interventions in rural Bangladesh and Kenya: the WASH Benefits study design and rationale. BMJ Open.

[29] Tofail F., Fernald L.C., Das K.K., Rahman M., Ahmed T., Jannat K.K. (2018). Effects of water quality, sanitation, handwashing, and nutritional interventions on child development in rural Bangladesh (WASH Benefits Bangladesh): a cluster-randomised controlled trial, Lancet Child Adolesc. Health.

[30] Luby S.P., Rahman M., Arnold B.F., Unicomb L., Ashraf S., Winch P.J. (2018). Effects of water quality, sanitation, handwashing, and nutritional interventions on diarrhoea and child growth in rural Bangladesh: a cluster randomised controlled trial. Lancet Glob. Health.

[31] Lin A., Ali S., Arnold B.F., Rahman M.Z., Alauddin M., Grembi J. (2020). Effects of water, sanitation, handwashing, and nutritional interventions on environmental enteric dysfunction in young children: a cluster-randomized, controlled trial in rural Bangladesh. Clin. Infect. Dis..

[32] Erhardt J.G., Estes J.E., Pfeiffer C.M., Biesalski H.K., Craft N.E. (2004). Combined measurement of ferritin, soluble transferrin receptor, retinol binding protein, and C-reactive protein by an inexpensive, sensitive, and simple sandwich enzyme-linked immunosorbent assay technique. J. Nutr..

[33] Namaste S.M., Aaron G.J., Varadhan R., Peerson J.M., Suchdev P.S. (2017). BRINDA Working Group, Methodologic approach for the Biomarkers Reflecting Inflammation and Nutritional Determinants of Anemia (BRINDA) project. Am. J. Clin. Nutr..

[34] Jung D.K., Tan S.T., Hemlock C., Mertens A.N., Stewart C.P., Rahman M.Z. (2023). Micronutrient status during pregnancy is associated with child immune status in rural Bangladesh. Curr. Dev. Nutr..

[35] Cogill B. (2003). https://www.scirp.org/reference/referencespapers?referenceid=285137.

[36] de Onis M., Onyango A.W., Van den Broeck J., Chumlea W.C., Martorell R. (2004). Measurement and standardization protocols for anthropometry used in the construction of a new international growth reference. Food Nutr. Bull..

[37] WHO. WHO Anthro Survey Analyser and other tools. [cited September 1, 2025]. https://www.who.int/tools/child-growth-standards/software.

[38] J. Coates, A. Swindale, P. Bilinsky, Household Food Insecurity Access Scale (HFIAS) for Measurement of Food Access: Indicator Guide, Version 3, Food and Nutrition Technical Assistance Project (FANTA), Washington, DC (2007)

[39] Leung K.C., Doyle N., Ballesteros M. (2003). Estrogen inhibits GH signaling by suppressing GH-induced JAK2 phosphorylation, an effect mediated by SOCS-2. Proc. Natl. Acad. Sci. U. S. A..

[40] Hotamisligil G.S., Erbay E. (2008). Nutrient sensing and inflammation in metabolic diseases. Nat. Rev. Immunol..

[41] Thorne-Lyman A.L., Fawzi W.W. (2012). Vitamin A and carotenoids during pregnancy and maternal, neonatal and infant health outcomes: a systematic review and meta-analysis. Paediatr. Perinat. Epidemiol..

[42] Schoos A.M.M., Vinther C., Nørgaard S., Brustad N., Stokholm J., Bønnelykke K. (2019). Environmental and genetic determinants of serum 25(OH)-vitamin D levels during pregnancy and early childhood. Children.

[43] World Health Organization, Guideline: Vitamin D Supplementation in Pregnant Women, WHO, Geneva, 2012 [cited September 1, 2025] https://www.ncbi.nlm.nih.gov/books/NBK310616/.

[44] Vestergaard AL, Justesen S, Volqvartz T, Aagaard SK, Andreasen MF, Lesnikova I, Uldbjerg N, Larsen A, Bor P. Vitamin D insufficiency among Danish pregnant women-Prevalence and association with adverse obstetric outcomes and placental vitamin D metabolism. Acta Obstet Gynecol Scand. 2021 Mar;100(3):480-488. 10.1111/aogs.14019. Epub 2021 Jan 12. PMID: 33030742. 33030742

