# Supplementary material for: Associations Between Micronutrient Status, Hormones, and Immune Status During Pregnancy and Child Growth in Rural Bangladesh: A Prospective Cohort Study
Source: Curr Dev Nutr. 2025 Nov 8;9(12):107596. doi: 10.1016/j.cdnut.2025.107596 (PMC12744257; doi:10.1016/j.cdnut.2025.107596)
Supplement: Multimedia component 1 [file mmc1.docx]

##

## Supplementary Table 1: Unadjusted vs. adjusted association between maternal estriol during pregnancy and child growth outcomes at 3, 14, and 28 months

| Maternal Estriol and Child Growth Status | Outcome | N | 25th Percentile | 75th Percentile | Outcome, 75th Percentile v. 25th Percentile | | | | | | | | | |
| --- | --- | --- | --- | --- | --- | --- | --- | --- | --- | --- | --- | --- | --- | --- |
|  |  |  |  |  | Unadjusted | | | | | Adjusted^^^ | | | | |
|  |  |  |  |  | Predicted Outcome at 25th Percentile | Predicted Outcome at 75th Percentile | Coefficient (95% CI) | P-value | FDR Corrected P-value | Predicted Outcome at 25th Percentile | Predicted Outcome at 75th Percentile | Coefficient (95% CI) | P-value | FDR Corrected P-value |
| Ln Estriol (ng/mL) | Length-for-Age Z-Score 3 months | 342 | 0.96 | 1.80 | -1.16 | -1.19 | -0.03 (-0.22, 0.17) | 0.79 | 0.79 | -1.41 | -1.29 | 0.12 (-0.04, 0.28) | 0.15 | 0.23 |
|  | Weight-for-Length Z-Score 3 months | 340 | 0.96 | 1.78 | -0.34 | -0.34 | 0 (-0.19, 0.19) | 0.99 | 0.99 | -0.19 | -0.23 | -0.04 (-0.25, 0.17) | 0.71 | 0.71 |
|  | Length-for-Age Z-Score 14 months | 521 | 0.75 | 1.71 | -1.31 | -1.40 | -0.09 (-0.19, 0.01) | 0.08 | 0.12 | -0.63 | -0.44 | 0.19 (0.03, 0.35) | 0.02 | 0.07^*^ |
|  | Weight-for-Length Z-Score 14 months | 521 | 0.75 | 1.71 | -0.92 | -1.07 | -0.15 (-0.24, -0.06) | 0.00 | 0.01^*^ | -0.83 | -0.91 | -0.08 (-0.2, 0.04) | 0.19 | 0.28 |
|  | Ln IGF-1 14 months (μg/L) | 427 | 0.72 | 1.71 | 38.12 | 37.34 | -0.79 (-3.25, 1.67) | 0.54 | 0.54 | 38.79 | 38.25 | -0.53 (-3.6, 2.53) | 0.75 | 0.75 |
|  | Length-for-Age Z-Score 28 months | 539 | 0.78 | 1.73 | -1.39 | -1.47 | -0.08 (-0.17, 0.01) | 0.07 | 0.12 | -1.45 | -1.44 | 0.01 (-0.13, 0.15) | 0.87 | 0.87 |
|  | Weight-for-Length Z-Score 28 months | 539 | 0.78 | 1.73 | -0.94 | -1.04 | -0.1 (-0.18, -0.02) | 0.02 | 0.02^*^ | -0.91 | -1.00 | -0.1 (-0.22, 0.03) | 0.12 | 0.28 |
|  | Ln IGF-1 28 months (μg/L) | 502 | 0.78 | 1.74 | 51.79 | 50.05 | -1.74 (-4.01, 0.54) | 0.13 | 0.27 | 53.57 | 50.28 | -3.29 (-5.9, -0.68) | 0.01 | 0.03^*^ |
| N, 25th Percentile, and 75th Percentile are from the unadjusted analyses.  ^^^Adjusted for the prespecified and prescreened covariates: child sex, child birth order, child gestational age, mother’s age, mother’s height, mother’s education, household food security, number of children < 18 y old in the household, number of people living in the compound, distance (in minutes) to the primary water source, household materials (wall, floor, roof), asset-based household wealth (electricity, wardrobe, table, chair or bench, khat, chouki, working radio, working black/white or color television, refrigerator, bicycle, motorcycle, sewing machine, mobile phone, land phone, number of cows, number of goats, number of chickens), and maternal exposure to intimate partner violence (IPV) during pregnancy and lifetime.  ^*^P < 0.1 after adjusting for multiple comparisons using the Benjamini-Hochberg procedure | | | | | | | | | | | | | | |

## Supplementary Table 2. Unadjusted vs. adjusted associations between maternal plasma cortisol during pregnancy and child growth outcomes at 3, 14, and 28 months

| Maternal Cortisol and Child Growth Status | Outcome | N | 25th Percentile | 75th Percentile | Outcome, 75th Percentile v. 25th Percentile | | | | | | | | | |
| --- | --- | --- | --- | --- | --- | --- | --- | --- | --- | --- | --- | --- | --- | --- |
|  |  |  |  |  | Unadjusted | | | | | Adjusted^^^ | | | | |
|  |  |  |  |  | Predicted Outcome at 25th Percentile | Predicted Outcome at 75th Percentile | Coefficient (95% CI) | P-value | FDR Corrected P-value | Predicted Outcome at 25th Percentile | Predicted Outcome at 75th Percentile | Coefficient (95% CI) | P-value | FDR Corrected P-value |
| Ln Cortisol (ug/dL) | Length-for-Age Z-Score 3 months | 342 | 2.67 | 3.29 | -1.20 | -1.15 | 0.05 (-0.23, 0.34) | 0.74 | 0.74 | -1.25 | -1.22 | 0.02 (-0.16, 0.21) | 0.82 | 0.99 |
|  | Weight-for-Length Z-Score 3 months | 340 | 2.66 | 3.29 | -0.31 | -0.33 | -0.03 (-0.23, 0.18) | 0.83 | 0.83 | -0.27 | -0.30 | -0.03 (-0.2, 0.15) | 0.77 | 0.82 |
|  | Length-for-Age Z-Score 14 months | 521 | 2.52 | 3.27 | -1.24 | -1.39 | -0.15 (-0.28, -0.01) | 0.03 | 0.09^*^ | -1.11 | -1.11 | 0 (-0.15, 0.14) | 0.99 | 0.99 |
|  | Weight-for-Length Z-Score 14 months | 521 | 2.52 | 3.27 | -0.92 | -1.06 | -0.14 (-0.26, -0.01) | 0.03 | 0.09 | -0.86 | -0.88 | -0.02 (-0.16, 0.12) | 0.82 | 0.82 |
|  | Ln IGF-1 14 months (μg/L) | 427 | 2.47 | 3.26 | 39.03 | 37.57 | -1.46 (-4.26, 1.34) | 0.31 | 0.31 | 39.16 | 38.05 | -1.12 (-3.99, 1.76) | 0.45 | 0.45 |
|  | Length-for-Age Z-Score 28 months | 539 | 2.53 | 3.27 | -1.36 | -1.47 | -0.1 (-0.22, 0.02) | 0.09 | 0.13 | -1.45 | -1.50 | -0.05 (-0.16, 0.06) | 0.37 | 0.99 |
|  | Weight-for-Length Z-Score 28 months | 539 | 2.53 | 3.27 | -0.93 | -1.03 | -0.1 (-0.21, 0.01) | 0.08 | 0.13 | -0.94 | -1.04 | -0.1 (-0.21, 0.01) | 0.08 | 0.24 |
|  | Ln IGF-1 28 months (μg/L) | 502 | 2.53 | 3.26 | 51.41 | 47.08 | -4.33 (-9.98, 1.32) | 0.13 | 0.27 | 51.84 | 48.59 | -3.25 (-8.52, 2.01) | 0.23 | 0.45 |
| N, 25th Percentile, and 75th Percentile are from the unadjusted analyses  ^^^Adjusted for prespecified and prescreened covariates: the covariates mentioned in Table 2.  *P < 0.1 after adjusting for multiple comparisons using the Benjamini-Hochberg procedure | | | | | | | | | | | | | | |

##

## Supplementary Table 3. Unadjusted vs. adjusted associations between maternal inflammation during pregnancy and child growth outcomes at 3, 14, and 28 months

| Maternal Inflammation and Child Growth Status | Outcome | N | 25th Percentile | 75th Percentile | Outcome, 75th Percentile v. 25th Percentile | | | | | | | | | |
| --- | --- | --- | --- | --- | --- | --- | --- | --- | --- | --- | --- | --- | --- | --- |
|  |  |  |  |  | Unadjusted | | | | | Adjusted^^^ | | | | |
|  |  |  |  |  | Predicted Outcome at 25th Percentile | Predicted Outcome at 75th Percentile | Coefficient (95% CI) | P-value | FDR Corrected P-value | Predicted Outcome at 25th Percentile | Predicted Outcome at 75th Percentile | Coefficient (95% CI) | P-value | FDR Corrected P-value |
| Ln AGP^1^ (g/L) | Length-for-Age Z-Score 3 months | 346 | -1.11 | -0.6 | -1.11 | -1.23 | -0.11 (-0.33, 0.1) | 0.31 | 0.7 | -1.13 | -1.33 | -0.2 (-0.41, 0.01) | 0.07 | 0.31 |
|  | Weight-for-Length Z-Score 3 months | 344 | -1.11 | -0.59 | -0.4 | -0.23 | 0.17 (0.02, 0.31) | 0.02 | 0.07^*^ | -0.45 | -0.07 | 0.38 (0.07, 0.69) | 0.02 | 0.07^*^ |
|  | Length-for-Age Z-Score 14 months | 526 | -1.11 | -0.54 | -1.32 | -1.31 | 0.01 (-0.26, 0.28) | 0.94 | 0.94 | -0.92 | -1.01 | -0.09 (-0.33, 0.15) | 0.47 | 0.64 |
|  | Weight-for-Length Z-Score 14 months | 526 | -1.11 | -0.54 | -1.05 | -0.89 | 0.16 (0.03, 0.29) | 0.01 | 0.07 | -0.91 | -0.82 | 0.08 (-0.03, 0.2) | 0.15 | 0.27 |
|  | Ln IGF-1 14 months (μg/L) | 431 | -1.14 | -0.54 | 36.76 | 38.57 | 1.81 (-0.56, 4.17) | 0.13 | 0.27 | 38.74 | 40.07 | 1.32 (-1.03, 3.67) | 0.27 | 0.41 |
|  | Length-for-Age Z-Score 28 months | 544 | -1.12 | -0.58 | -1.41 | -1.32 | 0.08 (-0.18, 0.35) | 0.54 | 0.81 | -1.41 | -1.43 | -0.03 (-0.23, 0.18) | 0.82 | 0.82 |
|  | Weight-for-Length Z-Score 28 months | 544 | -1.12 | -0.58 | -1.05 | -0.99 | 0.07 (-0.02, 0.15) | 0.13 | 0.27 | -0.99 | -0.94 | 0.05 (-0.03, 0.13) | 0.25 | 0.38 |
|  | Ln IGF-1 28 months (μg/L) | 507 | -1.14 | -0.58 | 48.26 | 51.11 | 2.85 (0.53, 5.17) | 0.02 | 0.1 | 50.95 | 53.52 | 2.58 (0.34, 4.81) | 0.02 | 0.14 |
|  |  |  |  |  |  |  |  |  |  |  |  |  |  |  |
| Ln CRP^2^ (mg/L) | Length-for-Age Z-Score 3 months | 346 | 0.11 | 1.5 | -1.3 | -1.06 | 0.24 (-0.08, 0.57) | 0.14 | 0.41 | -1.31 | -1.09 | 0.21 (-0.1, 0.53) | 0.19 | 0.34 |
|  | Weight-for-Length Z-Score 3 months | 344 | 0.12 | 1.51 | -0.36 | -0.4 | -0.04 (-0.42, 0.35) | 0.85 | 0.86 | -0.36 | -0.33 | 0.03 (-0.33, 0.39) | 0.88 | 0.88 |
|  | Length-for-Age Z-Score 14 months | 526 | 0 | 1.43 | -1.36 | -1.39 | -0.03 (-0.14, 0.09) | 0.67 | 0.86 | -1.13 | -0.96 | 0.16 (-0.05, 0.38) | 0.14 | 0.34 |
|  | Weight-for-Length Z-Score 14 months | 526 | 0 | 1.43 | -1.12 | -0.88 | 0.24 (0.03, 0.44) | 0.02 | 0.07 | -1.01 | -0.81 | 0.21 (0, 0.41) | 0.05 | 0.14 |
|  | Ln IGF-1 14 months (μg/L) | 431 | -0.12 | 1.41 | 37.11 | 38.35 | 1.24 (-1.18, 3.66) | 0.32 | 0.48 | 38.54 | 39.55 | 1.01 (-1.42, 3.45) | 0.42 | 0.42 |
|  | Length-for-Age Z-Score 28 months | 544 | -0.06 | 1.42 | -1.51 | -1.26 | 0.25 (0.02, 0.48) | 0.03 | 0.15 | -1.54 | -1.33 | 0.21 (0.01, 0.41) | 0.04 | 0.31 |
|  | Weight-for-Length Z-Score 28 months | 544 | -0.06 | 1.42 | -1.02 | -1.01 | 0.01 (-0.09, 0.11) | 0.86 | 0.86 | -0.96 | -0.95 | 0.01 (-0.09, 0.11) | 0.85 | 0.88 |
|  | Ln IGF-1 28 months (μg/L) | 507 | -0.09 | 1.41 | 49.58 | 50.46 | 0.88 (-2.06, 3.81) | 0.57 | 0.57 | 49.86 | 51.38 | 1.52 (-1.84, 4.88) | 0.38 | 0.42 |
|  |  |  |  |  |  |  |  |  |  |  |  |  |  |  |
|  |  |  |  |  |  |  |  |  |  |  |  |  |  |  |
| Sum score of 13 cytokines | Length-for-Age Z-Score 3 months | 285 | -0.69 | 0.65 | -1.36 | -1.23 | 0.13 (-0.22, 0.48) | 0.46 | 0.81 | -1.33 | -1.2 | 0.13 (-0.25, 0.52) | 0.51 | 0.64 |
|  | Weight-for-Length Z-Score 3 months | 283 | -0.7 | 0.65 | -0.3 | -0.35 | -0.05 (-0.25, 0.14) | 0.62 | 0.8 | -0.28 | -0.34 | -0.05 (-0.25, 0.14) | 0.59 | 0.76 |
|  | Length-for-Age Z-Score 14 months | 435 | -0.64 | 0.64 | -1.35 | -1.36 | -0.01 (-0.2, 0.18) | 0.93 | 0.94 | -1.1 | -1.17 | -0.07 (-0.32, 0.17) | 0.57 | 0.64 |
|  | Weight-for-Length Z-Score 14 months | 435 | -0.64 | 0.64 | -0.93 | -1.02 | -0.09 (-0.22, 0.03) | 0.15 | 0.27 | -0.83 | -0.94 | -0.12 (-0.24, 0.01) | 0.07 | 0.15 |
|  | Ln IGF-1 14 months (μg/L) | 366 | -0.63 | 0.64 | 36.41 | 35.45 | -0.96 (-3.8, 1.89) | 0.52 | 0.57 | 38.84 | 37.11 | -1.72 (-4.33, 0.89) | 0.2 | 0.4 |
|  | Length-for-Age Z-Score 28 months | 512 | -0.64 | 0.65 | -1.42 | -1.54 | -0.12 (-0.23, -0.01) | 0.03 | 0.15 | -1.32 | -1.49 | -0.17 (-0.4, 0.06) | 0.15 | 0.34 |
|  | Weight-for-Length Z-Score 28 months | 512 | -0.64 | 0.65 | -0.93 | -0.99 | -0.06 (-0.16, 0.04) | 0.24 | 0.36 | -0.86 | -0.96 | -0.1 (-0.14, -0.07) | 0 | 0^*^ |
|  | Ln IGF-1 28 months (μg/L) | 481 | -0.64 | 0.65 | 50.3 | 46.84 | -3.46 (-7.84, 0.91) | 0.12 | 0.27 | 50.99 | 47.27 | -3.72 (-8.1, 0.66) | 0.1 | 0.29 |
| AGP, alpha-1-acid glycoprotein  CRP, C-reactive protein  N, 25th Percentile, and 75th Percentile are from the unadjusted analyses  ^^^Adjusted for prespecified and prescreened covariates: the covariates mentioned in Table 2.  *P < 0.1 after adjusting for multiple comparisons using the Benjamini-Hochberg procedure | | | | | | | | | | | | | | |

## Supplementary Table 4. Unadjusted vs. adjusted associations between maternal micronutrients during pregnancy and child growth outcomes at 3, 14, and 28 months

| Maternal Micronutrients and Child Growth | Outcome | N | 25th Percentile | 75th Percentile | Outcome, 75th Percentile v. 25th Percentile | | | | | | | | | |
| --- | --- | --- | --- | --- | --- | --- | --- | --- | --- | --- | --- | --- | --- | --- |
|  |  |  |  |  | Unadjusted | | | | | Adjusted^^^ | | | | |
|  |  |  |  |  | Predicted Outcome at 25th Percentile | Predicted Outcome at 75th Percentile | Coefficient (95% CI) | P-value | FDR Corrected P-value | Predicted Outcome at 25th Percentile | Predicted Outcome at 75th Percentile | Coefficient (95% CI) | P-value | FDR Corrected P-value |
| Vitamin D (nmol/L) | Length-for-Age Z-Score 3 months | 346 | 32.62 | 53.37 | -1.14 | -1.24 | -0.09 (-0.26, 0.07) | 0.28 | 0.83 | -1.25 | -1.31 | -0.06 (-0.23, 0.11) | 0.49 | 0.67 |
|  | Weight-for-Length Z-Score 3 months | 344 | 32.62 | 53.47 | -0.33 | -0.26 | 0.07 (-0.2, 0.34) | 0.63 | 0.8 | -0.2 | -0.2 | 0 (-0.31, 0.3) | 0.99 | 0.99 |
|  | Length-for-Age Z-Score 14 months | 526 | 32.59 | 54.73 | -1.35 | -1.42 | -0.07 (-0.19, 0.05) | 0.26 | 0.83 | -0.89 | -0.96 | -0.07 (-0.18, 0.04) | 0.21 | 0.48 |
|  | Weight-for-Length Z-Score 14 months | 526 | 32.59 | 54.73 | -1.03 | -0.93 | 0.1 (-0.1, 0.31) | 0.34 | 0.75 | -0.85 | -0.77 | 0.08 (-0.12, 0.29) | 0.44 | 0.76 |
|  | Ln IGF-1 14 months (μg/L) | 431 | 32.48 | 54.53 | 37.57 | 37.04 | -0.53 (-3.7, 2.64) | 0.76 | 0.94 | 38.98 | 39.81 | 0.83 (-2.15, 3.81) | 0.6 | 0.77 |
|  | Length-for-Age Z-Score 28 months | 544 | 32.51 | 55.34 | -1.44 | -1.49 | -0.05 (-0.17, 0.08) | 0.46 | 0.86 | -1.41 | -1.48 | -0.06 (-0.19, 0.06) | 0.34 | 0.61 |
|  | Weight-for-Length Z-Score 28 months | 544 | 32.51 | 55.34 | -1.03 | -1 | 0.03 (-0.17, 0.22) | 0.79 | 0.89 | -0.85 | -0.81 | 0.04 (-0.18, 0.26) | 0.72 | 0.95 |
|  | Ln IGF-1 28 months (μg/L) | 507 | 32.59 | 55.53 | 49.92 | 53.32 | 3.4 (-2.3, 9.11) | 0.24 | 0.64 | 51.41 | 54.77 | 3.36 (-2, 8.71) | 0.22 | 0.44 |
|  |  |  |  |  |  |  |  |  |  |  |  |  |  |  |
| Vitamin D deficiency | Length-for-Age Z-Score 3 months | 346 | 0 | 0 | -1.22 | -1.22 | 0 (0, 0) |  |  | -1.35 | -1 | 0.35 (0, 0.7) | 0.05 | 0.18 |
|  | Weight-for-Length Z-Score 3 months | 344 | 0 | 1 | -0.27 | -0.48 | -0.22 (-0.56, 0.12) | 0.21 | 0.63 | -0.16 | -0.37 | -0.21 (-0.57, 0.15) | 0.26 | 0.68 |
|  | Length-for-Age Z-Score 14 months | 526 | 0 | 0 | -1.41 | -1.41 | 0 (0, 0) |  |  | -0.99 | -0.77 | 0.22 (0.01, 0.43) | 0.04 | 0.17 |
|  | Weight-for-Length Z-Score 14 months | 526 | 0 | 1 | -0.97 | -1.04 | -0.08 (-0.29, 0.14) | 0.5 | 0.8 | -0.79 | -0.83 | -0.04 (-0.26, 0.18) | 0.74 | 0.95 |
|  | Ln IGF-1 14 months (μg/L) | 431 | 0 | 1 | 37.98 | 37.31 | -0.67 (-5.11, 3.77) | 0.78 | 0.94 | 38.4 | 37.34 | -1.06 (-5.49, 3.37) | 0.65 | 0.77 |
|  | Length-for-Age Z-Score 28 months | 544 | 0 | 0 | -1.48 | -1.48 | 0 (0, 0) |  |  | -1.47 | -1.26 | 0.21 (0.03, 0.4) | 0.03 | 0.17 |
|  | Weight-for-Length Z-Score 28 months | 544 | 0 | 1 | -1.03 | -0.96 | 0.07 (-0.12, 0.26) | 0.46 | 0.8 | -0.92 | -0.84 | 0.08 (-0.11, 0.28) | 0.4 | 0.76 |
|  | Ln IGF-1 28 months (μg/L) | 507 | 0 | 1 | 50.09 | 50.37 | 0.29 (-4.92, 5.5) | 0.92 | 0.96 | 52.53 | 51.88 | -0.65 (-5.67, 4.36) | 0.81 | 0.81 |
|  |  |  |  |  |  |  |  |  |  |  |  |  |  |  |
| Ln RBP (μmol/L) | Length-for-Age Z-Score 3 months | 346 | 0.14 | 0.53 | -1.13 | -1.21 | -0.08 (-0.35, 0.2) | 0.6 | 0.86 | -1.3 | -1.28 | 0.01 (-0.16, 0.19) | 0.87 | 0.92 |
|  | Weight-for-Length Z-Score 3 months | 344 | 0.14 | 0.53 | -0.55 | -0.09 | 0.46 (0.16, 0.76) | 0 | 0.03^*^ | -0.39 | 0.07 | 0.46 (0.13, 0.78) | 0.01 | 0.05^*^ |
|  | Length-for-Age Z-Score 14 months | 526 | 0.13 | 0.52 | -1.36 | -1.37 | -0.02 (-0.14, 0.11) | 0.82 | 0.86 | -0.91 | -0.88 | 0.04 (-0.08, 0.15) | 0.56 | 0.67 |
|  | Weight-for-Length Z-Score 14 months | 526 | 0.13 | 0.52 | -1.08 | -0.91 | 0.16 (-0.01, 0.34) | 0.07 | 0.41 | -0.92 | -0.64 | 0.28 (0.09, 0.48) | 0 | 0.05^*^ |
|  | Ln IGF-1 14 months (μg/L) | 431 | 0.13 | 0.52 | 37.26 | 38.14 | 0.88 (-1.23, 2.99) | 0.42 | 0.72 | 37.72 | 39.5 | 1.78 (-0.33, 3.9) | 0.1 | 0.44 |
|  | Length-for-Age Z-Score 28 months | 544 | 0.12 | 0.51 | -1.41 | -1.39 | 0.02 (-0.17, 0.21) | 0.84 | 0.86 | -1.41 | -1.42 | -0.01 (-0.11, 0.1) | 0.92 | 0.92 |
|  | Weight-for-Length Z-Score 28 months | 544 | 0.12 | 0.51 | -1.07 | -0.91 | 0.16 (-0.04, 0.36) | 0.12 | 0.56 | -0.89 | -0.84 | 0.05 (-0.06, 0.17) | 0.4 | 0.76 |
|  | Ln IGF-1 28 months (μg/L) | 507 | 0.13 | 0.52 | 49.05 | 50.8 | 1.75 (-0.84, 4.34) | 0.19 | 0.64 | 51 | 52.65 | 1.65 (-0.65, 3.94) | 0.16 | 0.44 |
|  |  |  |  |  |  |  |  |  |  |  |  |  |  |  |
| Vitamin A deficiency^1^ | Length-for-Age Z-Score 3 months | 346 | 0 | 0 | -1.22 | -1.22 | 0 (0, 0) |  |  | -1.28 | -1.43 | -0.15 (-0.76, 0.47) | 0.65 | 0.73 |
|  | Weight-for-Length Z-Score 3 months | 344 | 0 | 1 | -0.3 | -0.45 | -0.15 (-0.77, 0.48) | 0.66 | 0.8 | -0.19 | -0.19 | 0 (-0.64, 0.64) | 0.99 | 0.99 |
|  | Length-for-Age Z-Score 14 months | 526 | 0 | 0 | -1.37 | -1.37 | 0 (0, 0) |  |  | -0.89 | -1.06 | -0.17 (-0.54, 0.2) | 0.38 | 0.62 |
|  | Weight-for-Length Z-Score 14 months | 526 | 0 | 1 | -0.99 | -0.9 | 0.08 (-0.29, 0.46) | 0.67 | 0.8 | -0.8 | -0.7 | 0.1 (-0.28, 0.48) | 0.62 | 0.93 |
|  | Ln IGF-1 14 months (μg/L) | 431 | 0 | 1 | 37.82 | 31.09 | -6.73 (-14.72, 1.25) | 0.1 | 0.64 | 39.83 | 30.84 | -8.99 (-17.09, -0.89) | 0.03 | 0.35 |
|  | Length-for-Age Z-Score 28 months | 544 | 0 | 0 | -1.44 | -1.44 | 0 (0, 0) |  |  | -1.42 | -1.68 | -0.26 (-0.59, 0.06) | 0.11 | 0.32 |
|  | Weight-for-Length Z-Score 28 months | 544 | 0 | 1 | -1.01 | -1.02 | -0.01 (-0.34, 0.32) | 0.96 | 0.96 | -0.92 | -0.95 | -0.03 (-0.36, 0.3) | 0.86 | 0.99 |
|  | Ln IGF-1 28 months (μg/L) | 507 | 0 | 1 | 50.15 | 50.42 | 0.27 (-9.11, 9.65) | 0.96 | 0.96 | 52.23 | 54.03 | 1.8 (-7.07, 10.66) | 0.7 | 0.77 |
|  |  |  |  |  |  |  |  |  |  |  |  |  |  |  |
|  |  |  |  |  |  |  |  |  |  |  |  |  |  |  |
| Ln sTfR (mg/L) | Length-for-Age Z-Score 3 months | 346 | 1.3 | 1.74 | -1.22 | -1.2 | 0.02 (-0.17, 0.21) | 0.86 | 0.86 | -1.34 | -1.24 | 0.1 (-0.09, 0.29) | 0.31 | 0.61 |
|  | Weight-for-Length Z-Score 3 months | 344 | 1.3 | 1.74 | -0.29 | -0.21 | 0.08 (-0.17, 0.32) | 0.56 | 0.8 | -0.14 | -0.15 | -0.01 (-0.26, 0.24) | 0.96 | 0.99 |
|  | Length-for-Age Z-Score 14 months | 526 | 1.29 | 1.71 | -1.15 | -1.48 | -0.34 (-0.61, -0.07) | 0.01 | 0.12 | -0.73 | -0.91 | -0.18 (-0.43, 0.06) | 0.14 | 0.36 |
|  | Weight-for-Length Z-Score 14 months | 526 | 1.29 | 1.71 | -0.96 | -0.98 | -0.02 (-0.26, 0.23) | 0.89 | 0.94 | -0.8 | -0.76 | 0.04 (-0.07, 0.16) | 0.46 | 0.76 |
|  | Ln IGF-1 14 months (μg/L) | 431 | 1.29 | 1.68 | 37.38 | 40.03 | 2.65 (-1.96, 7.26) | 0.26 | 0.64 | 38.12 | 40.71 | 2.59 (-1.87, 7.04) | 0.26 | 0.44 |
|  | Length-for-Age Z-Score 28 months | 544 | 1.3 | 1.71 | -1.4 | -1.43 | -0.03 (-0.25, 0.19) | 0.79 | 0.86 | -1.42 | -1.36 | 0.07 (-0.13, 0.26) | 0.51 | 0.67 |
|  | Weight-for-Length Z-Score 28 months | 544 | 1.3 | 1.71 | -1.02 | -0.92 | 0.09 (-0.12, 0.3) | 0.39 | 0.77 | -0.87 | -0.74 | 0.13 (-0.08, 0.33) | 0.22 | 0.68 |
|  | Ln IGF-1 28 months (μg/L) | 507 | 1.3 | 1.7 | 50.46 | 49.83 | -0.64 (-3.12, 1.84) | 0.63 | 0.94 | 50.63 | 53.53 | 2.9 (-1.81, 7.6) | 0.23 | 0.44 |
|  |  |  |  |  |  |  |  |  |  |  |  |  |  |  |
| Iron deficiency^1^ | Length-for-Age Z-Score 3 months | 346 | 0 | 0 | -1.21 | -1.21 | 0 (0, 0) |  |  | -1.31 | -1.2 | 0.11 (-0.21, 0.43) | 0.52 | 0.67 |
|  | Weight-for-Length Z-Score 3 months | 344 | 0 | 1 | -0.25 | -0.48 | -0.22 (-0.53, 0.08) | 0.15 | 0.56 | -0.15 | -0.35 | -0.2 (-0.54, 0.14) | 0.25 | 0.68 |
|  | Length-for-Age Z-Score 14 months | 526 | 0 | 0 | -1.31 | -1.31 | 0 (0, 0) |  |  | -0.98 | -1.19 | -0.21 (-0.4, -0.02) | 0.03 | 0.17 |
|  | Weight-for-Length Z-Score 14 months | 526 | 0 | 1 | -0.91 | -1.2 | -0.29 (-0.49, -0.09) | 0 | 0.03 | -0.79 | -1 | -0.21 (-0.4, -0.01) | 0.04 | 0.23 |
|  | Ln IGF-1 14 months (μg/L) | 431 | 0 | 1 | 38.15 | 36.24 | -1.91 (-6.04, 2.22) | 0.37 | 0.72 | 39.13 | 37.25 | -1.88 (-5.93, 2.17) | 0.37 | 0.55 |
|  | Length-for-Age Z-Score 28 months | 544 | 0 | 0 | -1.39 | -1.39 | 0 (0, 0) |  |  | -1.38 | -1.61 | -0.22 (-0.39, -0.06) | 0.01 | 0.16 |
|  | Weight-for-Length Z-Score 28 months | 544 | 0 | 1 | -0.99 | -1.09 | -0.09 (-0.27, 0.08) | 0.29 | 0.75 | -0.91 | -1.01 | -0.1 (-0.27, 0.07) | 0.26 | 0.68 |
|  | Ln IGF-1 28 months (μg/L) | 507 | 0 | 1 | 50.65 | 47.95 | -2.7 (-7.43, 2.03) | 0.27 | 0.64 | 53.21 | 49.8 | -3.41 (-7.93, 1.11) | 0.14 | 0.44 |
| RBP, retinol binding protein  sTfR, soluble transferrin receptor  N, 25th Percentile, and 75th Percentile are from the unadjusted analyses.  ^^^Adjusted for prespecified and prescreened covariates: the covariates mentioned in Table 2.  *P < 0.1 after adjusting for multiple comparisons using the Benjamini-Hochberg procedure  ^1^For binary exposure variables, 25^th^ and 75^th^ percentiles represent the distribution of the binary exposure (0/1) in the study population, while the predicted outcome represents the estimated child growth measurements for the exposure group. | | | | | | | | | | | | | | |
